# Cellular and Biochemical Characterization of Mesenchymal Stem Cells from Killian Nasal Polyp

**DOI:** 10.3390/ijms232113214

**Published:** 2022-10-30

**Authors:** Maria Mesuraca, Clelia Nisticò, Nicola Lombardo, Giovanna Lucia Piazzetta, Nadia Lobello, Emanuela Chiarella

**Affiliations:** 1 Laboratory of Molecular Haematopoiesis and Stem Cell Biology, Department of Experimental and Clinical Medicine, University “Magna Græcia”, 88100 Catanzaro, Italy; 2 Candiolo Cancer Institute, FPO-IRCCS, Department of Oncology, University of Torino, 10124 Candiolo, Italy; 3 Otolaryngology Head and Neck Surgery, Department Medical and Surgical Sciences, University “Magna Græcia”, 88100 Catanzaro, Italy

**Keywords:** Killian nasal polyp, mesenchymal stem cells, osteoblastic differentiation, adipocyte differentiation

## Abstract

Killian’s (antrochoanal) polyp is a unilateral nasal polypoid lesion of the maxillary sinus especially affecting children and young adults with unilateral nasal obstruction, pus discharge, and headache. Although its etiology is unclear, chronic inflammation, autoreactivity, allergies, and viral infections are implicated in its formation and development, causing nasal tissue remodeling. In this context, we isolated and cultured mesenchymal stem cells from surgical biopsies of three patients with Killian nasal polyp (KNP-MSCs) while healthy nasal tissue (HNT-MSCs) was used as control. Our results demonstrated that KNP-MSCs exhibited reduced cell proliferation compared to HNT-MSCs, and migrated less than the control, showing a partial epithelial phenotype with low mRNA levels of I-CAM and a significant increase of E-cad. Subsequently, both MSCs were induced to osteoblastic or adipocyte differentiation for up to 20 days. KNP-MSCs underwent to differentiate into osteoblasts but exhibited reduced ALP activity and calcium deposits and low mRNA levels of osteogenesis-associated genes compared to osteogenic induced-HNT-MSCs. Conversely, KNP-MSCs and HNT-MSCs have shown the same adipogenic differentiation potential, with a similar lipid droplet amount, adipocyte gene expression, and triacylglycerols content. Taken together, these results first demonstrated the cellular and molecular characterization of MSCs derived from the Killian nasal polyp.

## 1. Introduction

Nasal polyposis (NP) is a common benign and inflammatory lesion that originates from the mucosa of the nasal sinuses, resulting in the formation of a pedunculated mass. It affects about 4% of the population and is commonly present in patients with chronic rhinosinusitis (CRS). The etiology is still unclear, even if several associations with allergy, asthma, infection, cystic fibrosis, and aspirin sensitivity have been discovered. The most common symptoms are nasal obstruction, rhinorrhea, anosmia, post-nasal drip, and less frequent facial pain [1,2,3,4,5]. The main types of simple mucous nasal polyps are ethmoidal polyps and antrochoanal polyps, known as Killian polyps. Ethmoidal polyps are most frequently present in adults with multiple bilateral masses that arise from ethmoidal sinuses, uncinate process, middle turbinate, and middle meatus, expanding anteriorly toward the nares. Allergy and non-allergic rhinitis are associated with ethmoidal polyps genesis [2]. Immunological and genetic studies show that the molecular and cellular mechanisms driving the allergic inflammatory cascade involve multiple mediators and pathways as well as specific miRNAs; for example, miR-125b and miR-155 expression levels are significantly enhanced in NP patients, while miR-92a, miR-26b, miR-181b result are down-regulated [6,7,8].

Killian polyp is a non-neoplastic unilateral solitary polypoid lesion, described for the first time by Killian in 1906. It originates from the maxillary anthrum and extends through the nasal cavity to the choana, and it affects mainly children suffering from long-standing rhinosinusitis and rarely young adults, but also the elderly with epistaxis and pain, revealing in this cases malignant growth [2,8,9,10,11,12]. It is a unilateral pedunculated mass, composed of an antral portion, that is a central cystic cavity surrounded by edema and enclosed by a nasal/choanal portion of normal respiratory tissue, which emerges through an enlarged maxillary accessory ostium causing unilateral nasal obstruction pus discharge, headache; however, in rare cases, it can be bilateral, when the polyp is extremely bulky, manifesting a relevant nasal septum deviation [13].

Antrochoanal polyps are histologically characterized by firm fibrotic stroma showing high vascularization. They are mainly infiltrated with plasma cells and fewer eosinophils, unlike the histopathological structure of nasal polyps which is composed of stromal and subepithelial oedema as well as mixed inflammatory cell infiltration, of which more than 60% are eosinophils [14,15,16]. The recent guidelines (EPOS 2020) classified chronic rhinosinusitis with nasal polyps into primary and secondary forms. According to the anatomical extension, it is possible to differentiate localized and diffuse primary forms, which can be further defined on the basis of Type 2 vs. non-Type 2 etiopathogenetic mechanism. Regarding the first group, it is possible to differentiate localized and diffuse forms according to the anatomical extension and on the basis of Type 2 vs. non-Type 2 etiopathogenetic mechanism. Usually, bilateral polypod formations recognize an inflammatory cellularity that is oriented towards the prevalence of eosinophils in type 2 forms and neutrophils in non-type 2 forms [17,18]. The anthrochoanal polyp in this context must be framed in the context of a primary form of localized sinusitis, not type 2, and different anatomopathological studies exclude the presence of eosinophils, basophils, mast cells, Th2 lymphocytes, ILC2, and cytokines such as IL 4, IL5, IL13 which are instead present in Th2 polyps [19].

Despite its etiopathogenesis still being unknown, many studies have demonstrated that autoreactivity, allergies, and/or chronic inflammation, especially viral infections, such as the oncogenic human papillomaviruses HPV16, might play a crucial role in Kilian polyp development [11,20,21,22]. Clinically, nasal polyposis can be revealed by anterior rhinoscopy or nasal endoscopy. These examinations can be combined with imaging studies, such as computed tomography of the paranasal sinuses (PNS CT scan), in order to determine the severity of the disease and improve surgical planning [5]. Nasal polyp therapy can be different based on individual case assessment and involves a combination of observation, medical, and surgical treatments in order to reduce or completely eradicate it and improve symptoms.

Medical treatment requires the topical administration of nasal corticosteroids, delivered by drops or sprays, such as budesonide, fluticasone propionate, and mometasone furoate, to reduce the inflammation of the sinus mucosa, polyp size, and nasal congestion and improve quality of life. Additionally, in advanced or refractory cases associated with allergy, systemic steroids are administered to patients [1,5,23].

Furthermore, the statins, inhibitors of 3-hydroxy-3-methylglutaryl-CoA (HMG-CoA) reductase, generally utilized as cholesterol-lowering agents, have been proposed as a potential treatment for Th2-dominant nasal polyps [24,25]. Moreover, monoclonal antibodies targeting the IL-4 receptor, IgE, and IL-5 have been proposed as a novel and promising treatment for chronic rhinosinusitis with nasal polyposis (CRSwNP) [26,27]. Moreover, if the medical treatment is ineffective, it is necessary to resort to surgery, especially endoscopic sinus surgery (ESS). However, polyp recurrence is common with severe disease that affects 5–10% of patients [1,5,23].

Mesenchymal stem cells (MSCs) are stromal cells that have the ability to self-renew and also exhibit multi-lineage differentiation. MSCs can be isolated from a variety of tissues, such as the umbilical cord, endometrial polyps, menses blood, bone marrow, and adipose tissue, including nasal polyps from small surgical biopsies [28,29,30]. In recent decades, Mesenchymal stem cells (MSC) have attracted increasing attention for regenerative medicine thanks to their ability for self-renewal and differentiation into skeletal tissues such as osteoblasts and chondrocytes [31].

In the present study, we first investigated mesenchymal stem cells derived from three patients with Killian nasal polyps. The isolated mesenchymal multipotent progenitors exhibit many cell surface markers common to bone marrow mesenchymal stem cells (BMSCs), are capable of self-renewal, and display cell motility properties. We demonstrate, for the first time, that Killian nasal polyp-derived mesenchymal stem cells can differentiate into adipocytes and osteocytes, playing a key role in tissue healing and regenerative medicine.

## 2. Results

### 2.1. Molecular Characterization, Cell Viability, and Cell Cycle Analysis of Mesenchymal Stem Cells Derived from Killian Nasal Polyp

**Figure 1:**
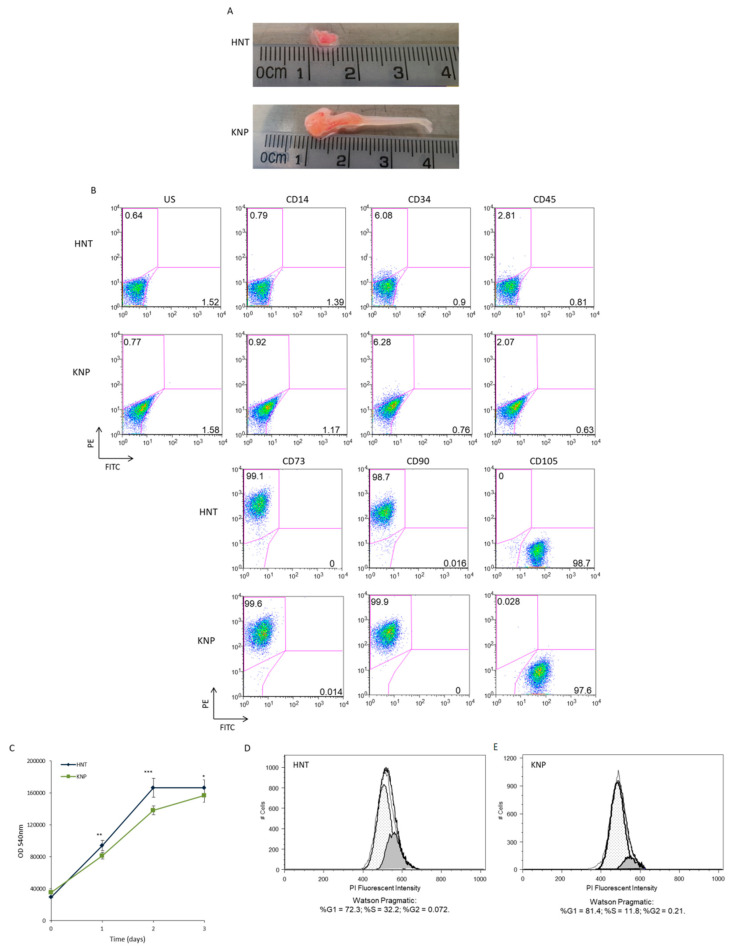
Characterization of mesenchymal stem cells isolated from Killian nasal polyp and healthy nasal tissue. (**A**) Representative nasal biopsies showing Killian nasal polyp and healthy nasal tissue from a patient. KNP and HNT MSCs were extracted by mechanical dissociation and collagenase digestion of related tissues. (**B**) Immunophenotypic characterization of KNP and HNT MSCs cells by flow cytometry analyzing CD73, CD90, CD105, CD29, CD14, CD34, and CD45 expression. (**C**) Cell viability of KNP and HNT MSCs were performed by 3-(4,5-dimethyl-2-thia-zolyl)-2, 5-diphenyl-2H-tetrazolium bromide (MTT) assay. (**D**) Cell cycle distribution was evaluated in KNP and HNT MSCs. (**E**) The percentage of KNP and HNT MSCs in G0/G1, S, and G2/M were determined by flow cytometry and quantified by Flow Jo analysis. Data are collected from three different experiments and presented as means ± SD (* *p* < 0.05; ** *p* < 0.001; *** *p* < 0.0001).

Killian polyps (KNP) were removed by polypectomy endoscopic surgery from three different patients, while the healthy nasal tissue (HNT) surrounding the sac-like growth was used as a control (Figure 1A).

Mesenchymal stem cells (MSCs) were isolated from both tissues, achieving clean monolayers after three weeks of culture. To assess mesenchymal phenotype of HNT-MSCs and KNP-MSCs isolated, FACS analysis was performed, showing that both cell types strongly expressed CD73, CD90, and CD105 mesenchymal markers and they were negative for CD14, CD34, and CD45 hematopoietic markers (Figure 1B and Figure S1A,B). Subsequently, HNT-MSCs and KNP-MSCs cell viability was monitored for three days by MTT assay, and we found that KNP-MSCs exhibited a low growth rate compared to HNT-MSCs in the three patients analyzed (Figure 1C). The fraction of actively dividing cells in KNP-MSCs was lower than HNT-MSCs; in particular, in KNP-MSCs was observed a decrease in the proportion of cells in the S phase (11.8%) of the cell cycle compared to control cells (32.2%) (Figure 1D). As expected, the majority of cells were in the G0/G1 phase of the cell cycle as emerged from histogram representation of cell-cycle distribution for HNT-MSCs (72.3%) and KNP-MSCs (81.4%), respectively (Figure 1E).

### 2.2. KNP-MSCs Show a Reduced Cell Migration Ability Compared to HNT-MSCs

**Figure 2:**
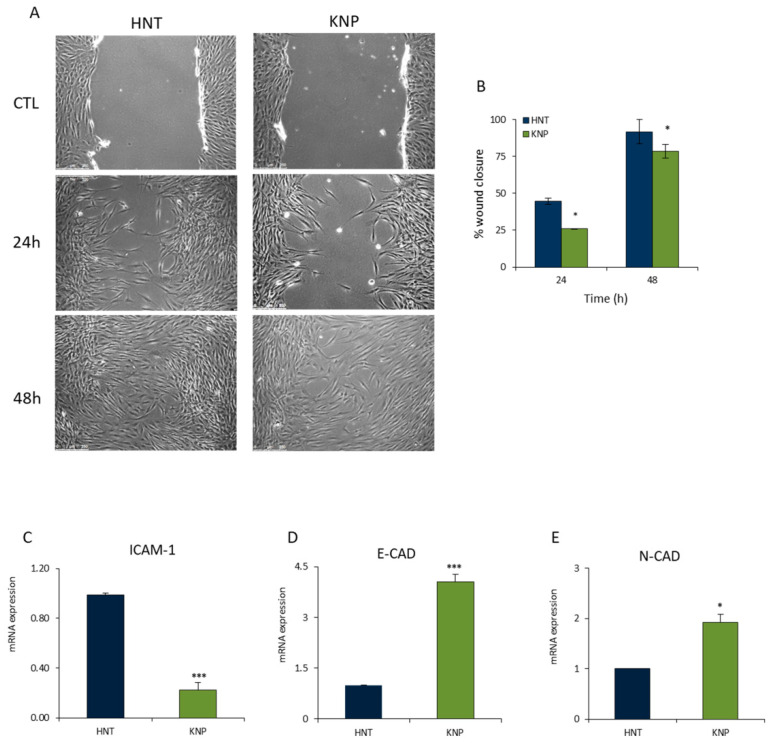
KNP-MSCs display decreased migration ability, keeping epithelial features compared to HNT-MSCs cells. (**A**) KNP-MSCs and HNT-MSCs migration was evaluated by scratch assay, capturing wound-healing pictures until 48 h, with a scale bar of 250 μm. (**B**) The bar plot displays the percentage of wound closure of KNP-MSCs and HNT-MSCs cells at 24 and 48 h, derived from ImageJ Fiji software analysis. (**C**–**E**) RT-qPCR was performed to compare EMT markers (ICAM-1-, E-CAD, N-CAD) expression of KNP-MSCs and HNT-MSCs cells. Data are shown as means ± SD derived from three different experiments (* *p* < 0.05; *** *p* < 0.0001).

MSCs have the ability to migrate and reach sites of injury and inflammation, stimulate tissue repair with endogenous progenitor cell activation and/or modulate immune responses. HNT-MSCs and KNP-MSCs migration ability was evaluated by scratch test, tracking live cells for 48 h (Figure 2A).

The migration rate, expressed as the percentage of wound closure, appeared slightly delayed in KNP-MSCs compared to HNT-MSCs over time, suggesting a reduction in the re-epithelialization process, especially after 24 h. At this time, the ability of KNP-MSCs to migrate was reduced by almost 35% compared to HNT-MSCs (Figure 2B). Furthermore, the ability to interfere with cell migration was proved by RT q-PCR analysis of related genes such as ICAM-1, E-CAD, and N-CAD. These findings revealed that KNP-MSCs were significantly lower in ICAM-1 at 24 h while exhibiting an increase of E-CAD more than N-CAD gene expression compared to HNT-MSCs, consistently with cadherin switching typical of the epithelium to mesenchymal transition processes (EMT) (Figure 2C–E).

### 2.3. Osteoblastic Differentiation Is Delayed in KNP-MSCs Compared to HNT-MSCs

**Figure 3:**
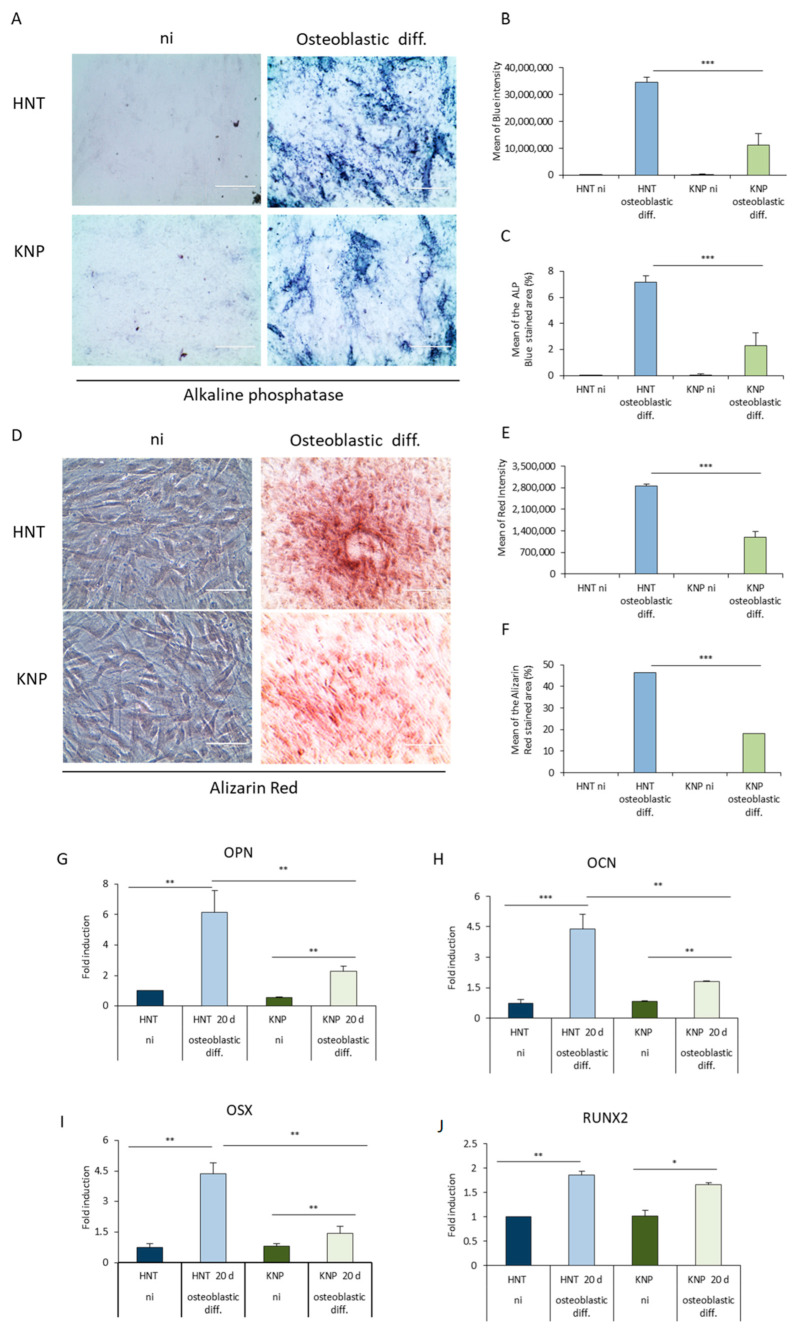
Delayed osteoblastic differentiation of KNP-MSCs compared to HNT-MSCs. (**A**) KNP-MSCs and HNT-MSCs were cultured in a standardized osteogenesis differentiation medium for up to 20 days. After seven days of osteogenic stimulation, all the samples were stained to detect Alkaline Phosphatase (ALP) activity and visualized under 20× magnification of phase contrast microscopy with a scale bar of 200 μm. (**B**,**C**) The mean of blue intensity and the percentage of blue stained area was quantified by ImageJ Fiji software. (**D**) Osteogenic differentiation of KNP-MSCs and HNT-MSCs was displayed by Alizarin Red staining after 20 days to visualize both intracellular and extracellular calcium deposits under 20× magnification of phase contrast microscopy with a Scale bar: 200 μm. (**E**,**F**) Cell calcium deposit quantification was performed by analyzing the red mean intensity and the percentage of Alizarin red-stained in triplicate area by ImageJ Fiji software. (**G**–**J**) RT-qPCR results reveal the relative mRNA expression of osteogenic-associated genes such as OPN, OCN, OSX, and RUNX2 after 20 days of osteogenic differentiation. The housekeeping gene GAPDH (Glyceraldehyde-3-phosphate dehydrogenase) was used to normalize gene expression results. All the results are shown as means ± SD from three different experiments (* *p* < 0.05; ** *p* < 0.001; *** *p* < 0.0001).

The ability of KNP-MSCs to differentiate into osteoblasts was investigated by cultivating cells in a standardized osteogenesis differentiation medium for up to 20 days. After seven days of osteogenic stimulation, Alkaline Phosphatase (ALP) activity was measured. The blue staining highlighted the cytoplasm of ALP-positive cells was less widespread and intense colored in KNP-MSCs stained compared to the osteogenic induced HNT-MSCs, demonstrating that KNP-MSCs were, however, able to differentiate slowly into osteoblasts (Figure 3A and Figure S2A–C).

The mean of blue intensity was used as a quantitative indicator of ALP protein expression and reflected the different distributions of the protein in the samples (Figure 3B and Figure S2A–C). Moreover, the percentage of the blue-stained area reveals a differentiation delay for KNP-MSCs. No signal was detected in the cells cultivated in the proliferation medium (Figure 3C). Subsequently, Alizarin Red S staining was performed to visualize both intracellular and extracellular calcium deposits, usually present in later stages of osteogenesis. The results have revealed that KNP-MSCs exhibited low calcium deposits compared to HNT-MSCs at 20 days of osteoblastic differentiation. Interestingly, Alizarin Red S binds to the calcium to form a typical red lake in HNT-MSCs, while this phenomenon is significantly attenuated in Killian nasal polyp-derived mesenchymal stem cells (Figure 3D and Figure S2A–C). These observations were confirmed by the analysis of the red mean intensity representing the number of stained calcium deposits in the cells. The percentage of Alizarin red-stained area in the KNP-MSCs was significantly lower than HNT-MSCs. There were no differences between the two cell populations not induced to differentiate into osteoblasts (Figure 3E,F). Considering the significantly low levels of ALP staining and the less mineralized nodules generated in KNP-MSCs induced to osteoblastic differentiation, the analysis of osteogenic differentiation-related genes was performed after 20 days of stimulation. The transcript levels of selected osteogenic-associated genes such as Osteopontin (OPN), Osteocalcin (OCN), Osterix (OSX), and Runt-related transcription factor 2 (RUNX2) revealed a reduced ability for KNP-MSCs to differentiate into osteoblasts compared to mesenchymal stem cells derived from healthy tissue (Figure 3G–J).

### 2.4. KNP-MSCs Are Able to Differentiate into Adipocytes Similarly to HNT-MSCs

**Figure 4:**
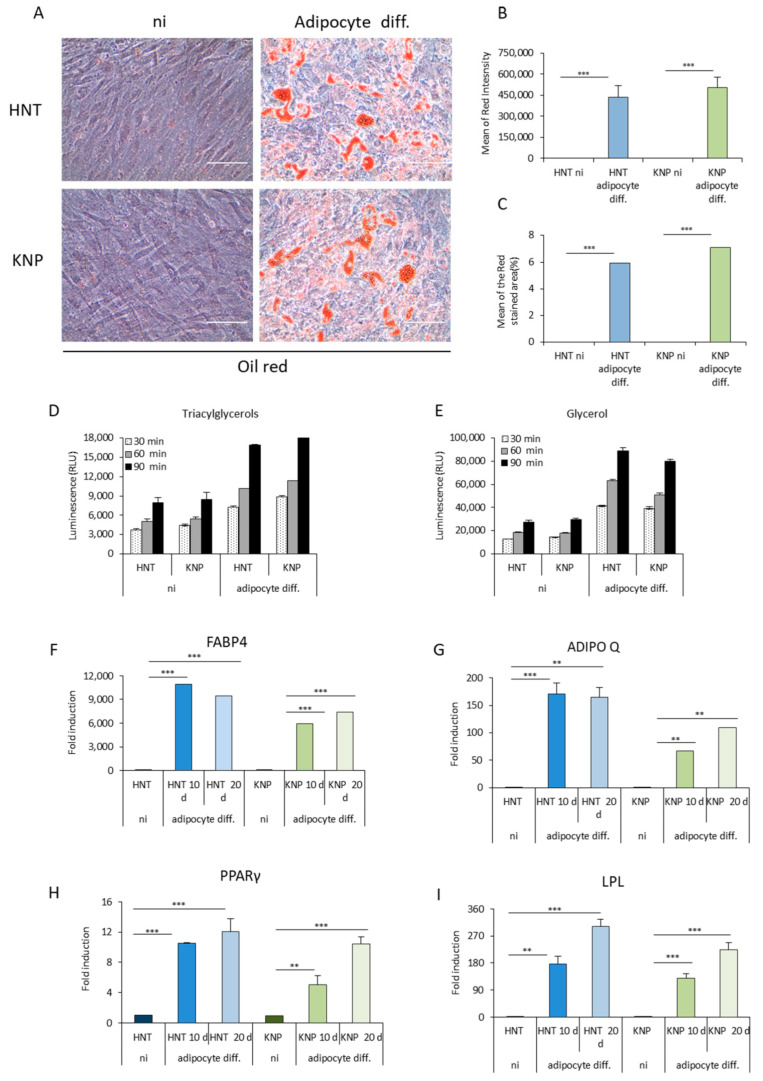
Adipocyte differentiation of KNP-MSCs and HNT-MSCs. (**A**) KNP-MSCs and HNT-MSCs were cultured with a standardized adipogenesis differentiation medium and, after 20 days, stained with Oil Red O to detect lipid droplet amount under 40× magnification of phase contrast microscopy. Scale bar: 100 μm. (**B**,**C**) ImageJ Fiji software analyses were performed to quantify the percentage of red stained area and the mean of red intensity after 20 days of adipogenic differentiation. (**D**,**E**) Triglyceride-Glo™Assay was performed to assess triacylglycerols and glycerol levels after 20 days of adipogenic differentiation. (**F**–**I**) The adipogenic differentiation-related gene expression of PPARγ2, FABP4, Adipo-Q, and LPL were evaluated by RT-qPCR after 10 and 20 days in adipogenic differentiation medium, using the housekeeping gene GAPDH for normalization. Data are displayed as means ± SD from three different experiments (** *p* < 0.001; *** *p* < 0.0001).

In order to study the KNP-MSCs plasticity to differentiate into adipocytes, we cultured KNP-MSCs and HNT-MSCs with a standardized adipogenesis differentiation medium. After 20 days, a similar lipid droplet amount was detected in both cell lines by Oil Red O staining (Figure 4A), and this result was confirmed by analyzing the percentage of the red-stained area and the mean of red intensity (Figure 4B).

In addition, the non-polar lipids content in lipid droplets was measured by Triglyceride-Glo™Assay. The number of triacylglycerols and glycerol that is released from an enzymatic reaction with a lipase was similar in KNP-MSCs and HNT-MSCs induced to differentiate into adipocytes for 20 days (Figure 4C,D). The greatest levels of triacylglycerols were observed after a lipase incubation time of 90 min, while base levels are detectable in non-induced cells. Based on previous results, the analysis of the main adipogenic differentiation-related genes such as PPARγ2 (peroxisome proliferator-activated receptors), FABP4 (fatty acid-binding protein 4), Adipo-Q (adiponectin), and LPL (lipoprotein lipase) was performed by RT-qPCR after 10 and 20 days in the differentiation medium. The levels of the considered mRNAs progressively increased in KNP-MSCs and HNT-MSCs stimulated to differentiate into adipocytes compared to undifferentiated control cells, respectively. At the same time, FABP4, ADIPO-Q, and LPL transcript levels in adipogenic-induced KNP-MSCs were slightly lower than in adipogenic-induced HNT-MSCs, while PPARγ2 expression is similar in both adipogenic-induced cell types (Figure 4E–H).

## 3. Discussion

Nasal polyps represent an alternative source of mesenchymal stem cells and non-hematopoietic stromal cells with the capacity for self-renewal and multi-lineage differentiation potential. These multipotent progenitor cells play a critical role in tissue repair and regeneration and exhibit unique immunomodulatory properties [4,23,32,33]. Mesenchymal stem cells (MSC) can be isolated from several adult human tissues, including bone/bone marrow, fat, Wharton’s jelly, umbilical cord blood, placenta, periodontal ligament, and pancreas [34]. In this study, we first examined the availability of Killian nasal polyps as a source of MSCs.

In particular, we isolated and cultivated mesenchymal stem cells (KNP-MSC) from three patients with antrochoanal polyp, a rare and benign neoplasm that arises from the maxillary sinus, most often on the posterior wall, giving rise to a pedunculated, painless white structure that expands into the nasal cavity [35,36]. In the long term, the edematous nasal mucosa lining the maxillary sinus obstructs the entire nasal cavity, producing the typical symptoms associated with irritation and swelling (inflammation) [37,38]. Here, we provide a cellular and molecular description of Killian nasal polyp derived-mesenchymal stem cells (KNP-MSCs). These cells show a fibroblast-like appearance with the ability of plastic plate adherence and possess proliferative potential because they can be effectively expanded in vitro. In addition, we demonstrated by flow cytometry analysis that KNP-MSCs express the minimal phenotypic pattern required for the identification of MSCs by being immunopositive for CD73, CD90, and CD105 expression, while results were negative for the haematopoietic cell surface antigens CD14, CD34, and CD45. These features make Killian nasal polyp a rich source of MSCs, according to the criteria established by the International Society for Cellular Therapy [39]. Furthermore, MSCs from various tissue sources can exhibit different biological characteristics related to cell proliferation or differentiation potential [40]. In particular, KNP-MSCs cells have shown a lower proliferative rate compared to HNT-MSCs, caused by induction in the percentage of cells in the G0/G1 phase and a consequent S peak decrease.

Epithelial to mesenchymal transition is a typical feature of chronic rhinosinusitis with nasal polyps (CRSwNP), in which epithelial cells acquire a mesenchymal phenotype by losing their cell polarity and cell–cell adhesion and gaining migratory and invasive properties [4,41]. The migratory ability appears preserved in the cell population derived from antrochoanal polypoid lesions originating and sustaining from a type 1 inflammation pattern, as emerged from the data presented here (Figure 2A–E).

Importantly, we demonstrated that KNP-MSCs are able to move and are involved in dynamic reparative processes following tissue injury in vitro. However, KNP-MSCs exhibited a delayed EMT program compared to HNT-MSCs to such an extent that wound healing was not complete after 48 h. The larger time required for the closure of the space between wounds in KNP-MSCs could explain the reason because this type of polyp recurs much less than nasal polyps related to type 2 inflammation. In addition, cell adhesion molecules are crucial markers of inflammation and fibrosis [19]. In KNP-MSCs, the epithelial-mesenchymal transition (EMT) process results in a transcriptionally reprogramming whereby one key change is the low expression of ICAM-1 and high levels of E-cadherin mRNA; the levels of N-cadherin transcript are also relatively high suggesting that these cells have a mixed epithelial and mesenchymal phenotype.

Mesenchymal Stem Cells (MSC) are multipotent cells able to differentiate in vitro into adipocytes, chondrocytes, osteoblasts, myocytes, and ß-pancreatic islet cells, under specific stimuli [42,43,44]. By their multi-lineage potential, MSCs contribute to the regeneration of mesenchymal tissues, such as bone, cartilage, muscle, ligament, and tendon, highlighting a relevant role in regenerative medicine [45]. Here, we investigated the multipotency ability of KNP-MSCs by differentiating cells into osteoblasts and adipocytes in vitro.

In particular, we found that KNP-MSCs are capable of differentiating into osteoblasts, although at a lower level compared to mesenchymal stem cells derived from healthy tissue.

However, KNP-MSCs can differentiate into functional matrix-synthesizing osteoblasts in a process that progresses through three stages: proliferation, matrix maturation, and mineralization [46]. KNP-MSCs secrete a considerable amount of alkaline phosphatase in their cytoplasm when cultured in an osteoblastic differentiation medium for one week, while the formation of calcified nodules, a late sign of late osteoblastic differentiation, is evident after 21 days. In addition, the osteoblastic-related genes OSX, RUNX2, OCN, and OPN are weakly expressed in uncommitted mesenchymal cells, and their expression is upregulated in preosteoblasts, reaching the maximal level in mature osteoblasts [47,48].

The sequential process of osteoblasts formation appears delayed in KNP-MSCs compared to HNT-MSCs, in which the overexpression of RUNX2 is similar, but the mRNA levels of OSX, OPN, and OCN are downregulated compared to the osteogenic-induced HNT-MSC, suggesting the presence of a small pool of more immature mesenchymal stem cells from this alternative source [49,50].

Concomitantly, we found that KNP-MSCs are able to commit to the adipose lineage and differentiate into mature adipocytes.

Oil Red O staining for intracellular lipid droplet accumulation in MSCs derived from Killian nasal polyps resulted similarly to that of healthy nasal tissue after 21 days under adipogenic differentiation, confirming their profile of multipotential stem/progenitor cells.

In particular, during KNP-MSCs adipogenic differentiation, triglycerides accumulate in lipid droplets, becoming increasingly similar to HNT-MSCs. Triacylglycerol content increased equally both in KNP-MSCs and HNP-MSCs in a time-dependent manner, as emerged by measuring glycerol generated by triacylglycerol lipase enzymatic reaction [51,52]. As expected, the expression of fat marker genes (such as Fabp4, Adipoq, peroxisome proliferator-activated receptor gamma (PPARγ), and LPL), normally associated with adipocyte differentiation, reflected high transcript levels in both mesenchymal cell populations compared to control cells over the time [53]. Whereas mesenchymal stem cells derived from nasal polyp with chronic rhinosinusitis type 2 undergo apoptosis when cultured for five days in the differentiation medium, these data indicate that KNP-MSCs showed greater adipogenic potential [30].

In conclusion, according to our research, Killian nasal polyps contain a pool of adult mesenchymal stem cells with self-renewal ability, migratory properties, and the capability to differentiate into osteoblasts and adipocytes. This evidence paves the way for experimental studies aimed at understanding the cellular and molecular bases of antrochoanal polypoid lesions as well as potential new therapeutic implications.

However, further studies are required to understand the immunomodulatory effects of KNP-MSCs and their involvement in the Killian nasal polyp origin and evolution [54,55]. Therefore, Killian nasal polyps could be considered an alternative source of therapeutically useful MSCs for the treatment of immune-mediated diseases.

## 4. Materials and Methods

### 4.1. HNT and KNP Mesenchymal Stem Cells Isolation and Culture

KNP MSCs cells were obtained by polypectomy endoscopic surgery of three different patients (0.7–0.9 cm) and healthy nasal tissue (HNT) MCSs were acquired from each patient and used as control (0.2–0.3 cm). Cells were isolated by mechanical dissociation, a scalpel blade, followed by enzymatic digestion with Collagenase IV 1 mg/mL for 4–12 h at 37 °C. Cells were washed in PBS (Phosphate-buffered saline) and filtered through 70 µm filters, and adherent cells were cultivated in tissue culture treated 6 wells plates in MesenPRO RSTM medium (Thermo Fisher Scientific, Milan, Italy), 1× Glutamax (Life Sciences, Monza, Italy), 50U of penicillin/50 μg streptomycin/mL and gentamicin 20 μg/mL and then incubated at 37 °C in 5% CO_2_. After three passages, a uniform monolayer of adherent cells was obtained. Informed consent was obtained from patients.

### 4.2. Flow Cytometry and Cell Cycle Analysis

The immunophenotype of HNT and KNP-MSCs was analyzed by flow cytometry. 1 × 10^3^ cells were stained for 30 min, in the dark, at 4 °C with fluorescent-conjugated antibodies specific for the mesenchymal markers, PE-CD29, PE-CD73, PE-CD90, and FITC-CD105, or for the hematopoietic markers, PE-CD14, PE-CD34, and PE-CD45 (Miltenyi Biotec S.r.l., Bologna, Italy) [56]. The cells were then washed, re-suspended in 300 μL of PBS and acquired on the BD FACscan™ II. Data were analyzed with FlowJo software 8.8.6. For cell cycle analysis, 2 × 10^5^ cells were fixed in ice-cold 70% ethanol for 30 min, in the dark, at 4 °C and then were washed three times in cold PBS. Subsequently, the cells were treated with RNase (40 μg/mL) (Sigma-Aldrich, Milan, Italy) for 30 min at room temperature and then stained with PI (50 μg/mL) for 10 min at room temperature. Analysis was performed by BD FACscan™ II, using 488-nm excitation. The data have been processed by FlowJo software 8.8.6 with histograms representing the DNA content distribution across the steps of the cell cycle.

### 4.3. Cell Proliferation Assay

MTT assay was used to determine MSCs cell proliferation. 1 × 10^3^ cells were seeded in 96-well plates with 100 μL of growth media and then cultured for 3 days [57]. Every day MTT solution (10 μL from stock solution of 5 mg/mL) (Sigma Aldrich, Milan, Italy) was added to the cell culture medium and incubated at 37 °C for 90 min. Finally, the dye crystals were dissolved with 0.08 N HCl, and the absorbance was measured at 590 nm using the spectrophotometer Glomax (Promega, Milan, Italy). The absorbance values registered were directly proportional to the number of viable MSCs cells.

### 4.4. Migration Assay

The ability of KNP-MSCs cell migration was determined by wound healing assay. Briefly, 5 × 10^5^ cells were seeded in 6 well plates and incubated overnight at 37 °C. The next day a gap on a cell monolayer was created, and the cells were washed gently with PBS and cultured with fresh complete medium. The recolonization of the scratched region was followed and imaged every 24 h at 20× magnification for a total of 48 h using a DFC 3000 G camera mounted on a Leica microscope (DM IL LED, Leica Microsystems Srl, Milan, Italy). The percentage of wound closure was obtained by subtracting gaps’ areas at 24, 30, and 36 h post-wounding from gaps’ areas at 0 h.

### 4.5. Osteoblastic Differentiation

For osteoblastic differentiation, 5 × 10^3^ cells were cultured in StemPro osteogenesis differentiation medium for 20 days (cat. No. A1007201 Life Technologies). The osteogenic medium was changed every three days. Control cells were cultured in MesenPro RSTM medium (Life Technologies, Milan, Italy).

After six days of culture in the differentiation medium, ALP Activity was detected using BCIP-NBT staining. Firstly, cells were washed twice in PBS and incubated with BCIP/NBT Blue Liquid Substrate solution (B3804 Sigma-Aldrich) in the dark for 10 min at room temperature. The enzymatic reaction was blocked by washing the cells twice with distilled water. The intensity of ALP staining was visualized under 10× magnification bright-field microscopy using an EVOS M5000 Cell Imaging System (Life Technologies). The images were quantified for Blue-stained area by an ImageJ macro [58]. Mature osteoblasts were identified by staining with Alizarin Red S (Sigma) after 20 days of osteoblastic differentiation. Briefly, cells were fixed in 10% formalin for 20 min at room temperature, washed three times with PBS, and then stained with Alizarin Red solution for 50 min at room temperature. After three washes in distilled water, cells were analyzed under the Evos bright-field microscope (EVOS M5000 Cell Imaging System, Life Technologies), and the images were processed by an ImageJ macro [59]. 

### 4.6. Adipogenic Differentiation

To induce adipogenic differentiation, 1 × 10^4^ cells/cm2 were seeded in 12-well plates and incubated for 20 days with STEM PRO^®^ Adipogenesis differentiation medium (cat. No. A1007001 Life Technologies). The adipocyte induction medium was changed every three days. Control cells were maintained in culture in MesenPro RSTM medium (Life Technologies). Following adipogenic stimulation, cytoplasmic lipid droplets were stained with Oil red O. Briefly, the cells were washed with PBS twice and then fixed with pre-cooled 10% formaldehyde for 15 min at −20 °C. After being washed twice with PBS, to remove all the formaldehyde, cells were stained for 30 min at room temperature in freshly diluted Oil Red O Solution 0.5% in isopropanol diluted (3 parts oil red O stock and 2 parts distilled water), filtered with a 0.45 µm filter (O1391-Sigma). After two PBS washes, images were acquired using a bright-field microscope (EVOS M5000 Cell Imaging System, Life Technologies). The percentage and the intensity of the red-stained area were quantified by an ImageJ macro [60].

### 4.7. Triacylglycerol Assay

Triacylglycerols content was determined using the Triglyceride-Glo™ Assay (Promega) according to the manufacturer’s instructions. Relative triacylglycerol level was expressed as normalized luminescence.

### 4.8. RNA Isolation, Reverse Transcription, and Quantitative RT-PCR

RNA extraction was performed by using TRI REAGENT™ according to the manufacturer’s instructions (Sigma-Aldrich Technical Bulletin MB-205). RNA was quantified with the NanoDrop 2000/2000c Spectrophotometer (Thermo Fisher Scientific), and the overall quality of the preparation was assessed by electrophoresis on 1.5% agarose gels run in MOPS buffer, pH 7.1 (0.4 M MOPS (3-(N morpholino)propanesulfonic acid), 0.1 M NaAc, 20 mM EDTA (Ethylenediaminetetraacetic acid)), and 10% formaldehyde.

cDNA was then synthesized from 1 ug of RNA by using SuperScript III reverse transcriptase at 42 °C and 2.5 µM random hexamers (Life Technologies). Quantitative RT PCR was carried out using the SYBR™ Green master mix (Bio-Rad, Milan, Italy) and the iQ5 multicolor detection system (Bio-Rad). Primer sequences are listed in Table 1. The PCR cycling conditions included an initial denaturation of 95 °C for 3 min followed by 45 cycles of 95 °C for 10 s, 60 °C for 10 s, and 72 °C for 20 s, followed by a melting curve. All mRNA levels were normalized to GAPDH expression, and the relative gene expression was calculated using the comparative threshold cycle Ct method [61].

### 4.9. Statistical Analysis

Student’s *t*-test was used to calculate all *p* values. Asterisks in the figures indicate the statistically significant difference: (* *p* < 0.05; ** *p* < 0.001; *** *p* < 0.0001).

## 5. Study Limitation

Publication limitation could be represented by a small number of samples, only three patients. However, Killian nasal polyp is classified as an infrequent, benign lesion usually arising from the posterior, inferior, lateral, or medial walls of the maxillary antrum and represents only 4–6% of all nasal polyps in adults. Considering incidence data, the collection of samples from Killian nasal polyp results is very difficult. Here we first characterized the mesenchymal stem cells component of the anthrocoanal polyp; however, increasing the number of samples would minimize common bias and uniformly define the MSCs potential from this rare type of nasal polyp.

## Figures and Tables

**Table 1:** List of Primers for RT qPCR analysis.

Gene	Forward Primer (5′→3′)	Reverse Primer (5′→3′)
GAPDH	CACCATCTTCCAGGAGCGAG	TCACGCCACAGTTTCCCGGA
ICAM-1	CCTTCCTCACCGTGTACTGG	TGGCTCCCGTTTCAGCTCCT
N-CAD	CCGCGGCCCGCTATTTGTCA	CCAGAAGCCTCTACAGACGCCTGA
E-CAD	TACGCCGGGACTCCACCTA	CCAGAAAGCGAGGCCTGAT
PPARγ2	CCTATTGACCCAGAAAGCGATT	CATTACGGAGAGATCCACGGA
FABP4	TGGGCCAGGAATTTGACGAA	GACGCATTCCACCACCAGTT
ADIPO-Q	AGGGTGAGAAAGGAGATCC	GGCATGTTGGGGATAGTAA
OSX	CCCAGGCAACACCCTACTC	GGCTGGATTAAGGGGAGCAAA
OPN	CTCCATTGACTCGAACGACTC	CAGGTCTGCGAAACTTCTTAGAT
RUNX2	GGAGTGGACGAGGCAAGAGTTT	AGCTTCTGTCTGTGCCTTCTGG

## Data Availability

Not applicable.

## Supplementary Materials

The supporting information can be downloaded at: https://www.mdpi.com/article/10.3390/ijms232113214/s1.
