# Supplementary figures and images for: Cellular and Biochemical Characterization of Mesenchymal Stem Cells from Killian Nasal Polyp
Source: Int J Mol Sci. 2022 Oct 30;23(21):13214. doi: 10.3390/ijms232113214 (PMC9656559; doi:10.3390/ijms232113214)

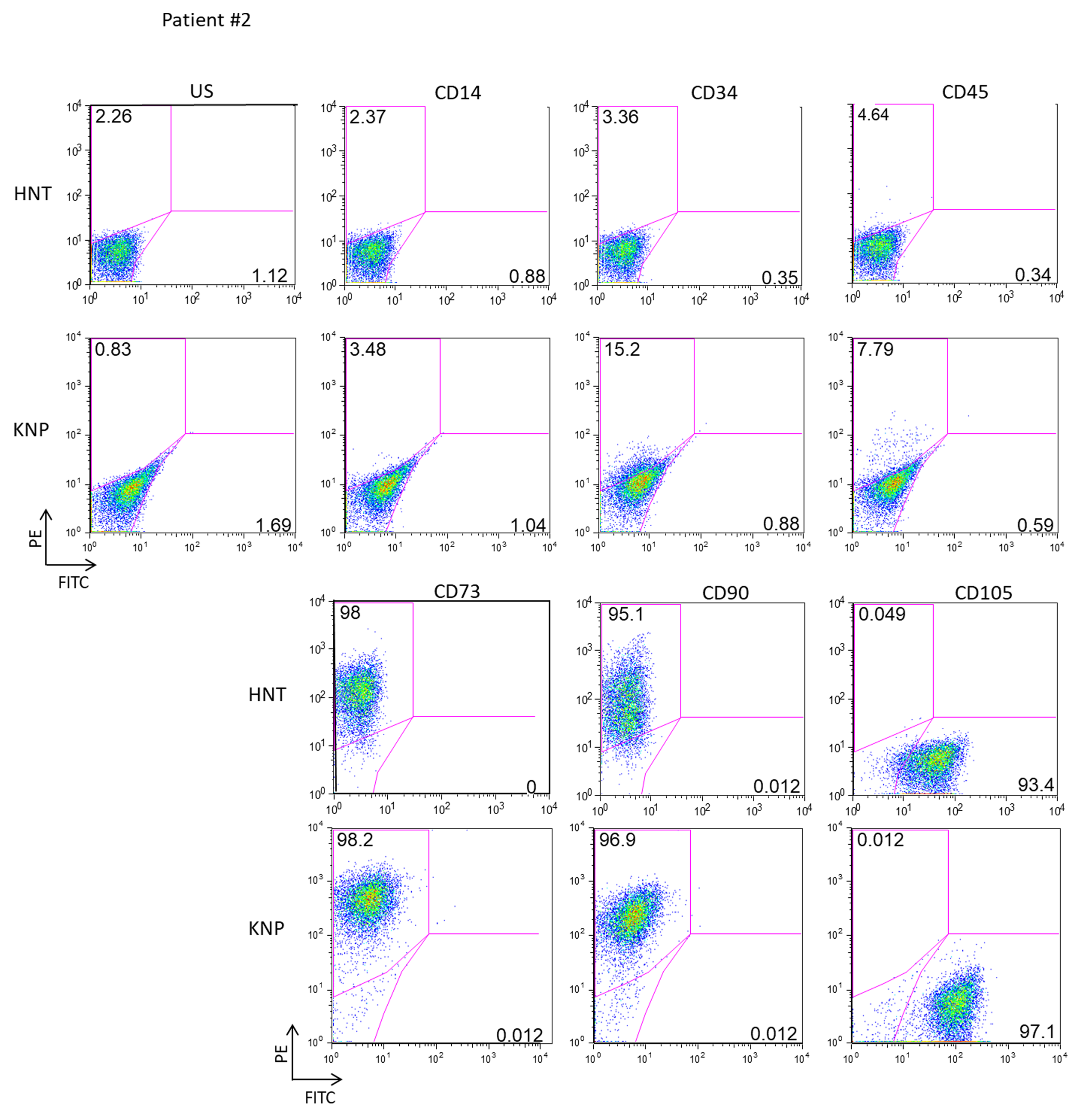

Supplement: Supplementary file 1 [file ijms-23-13214-s001.zip › S1A.tif]

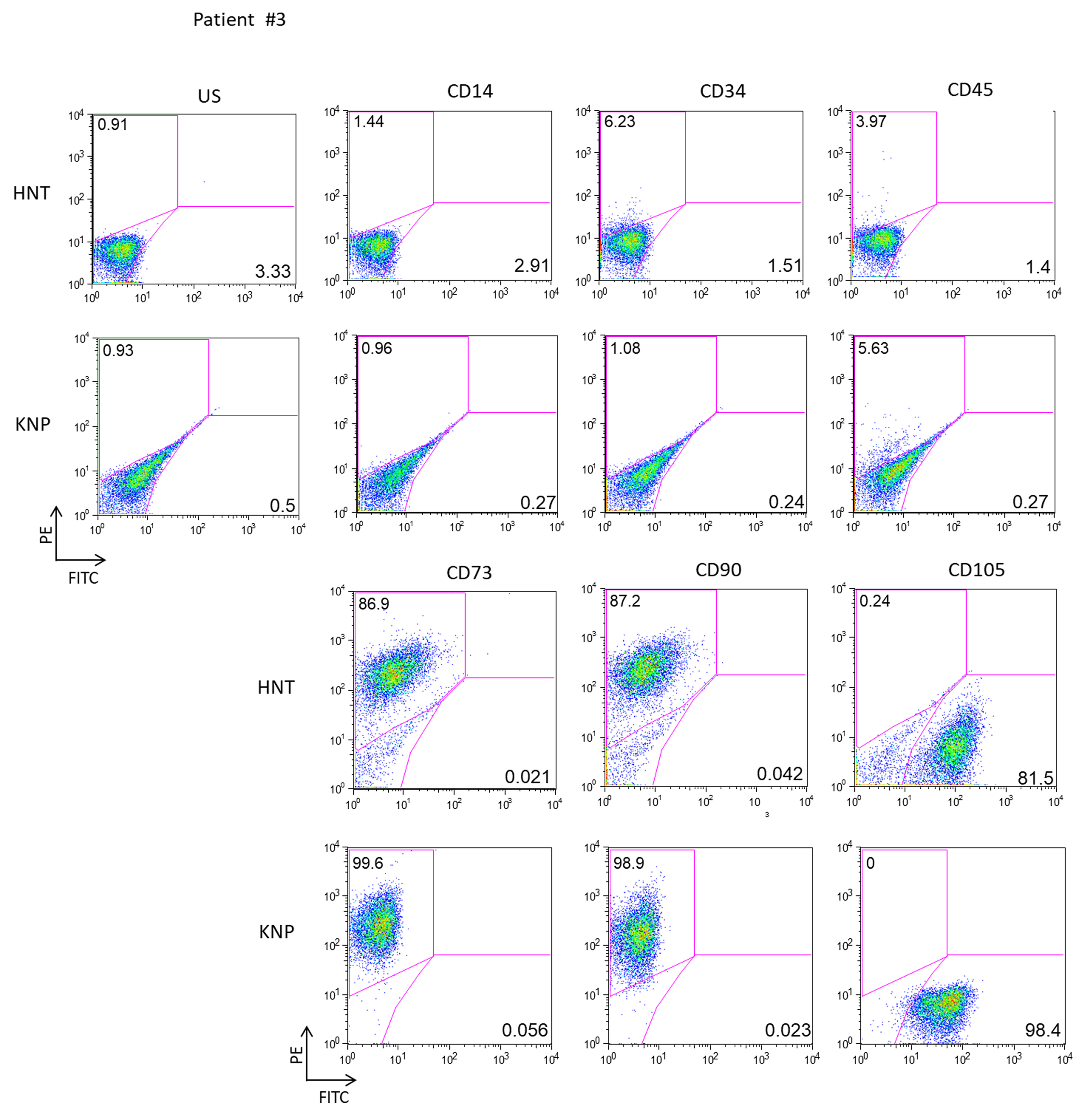

Supplement: Supplementary file 1 [file ijms-23-13214-s001.zip › S1B.tif]

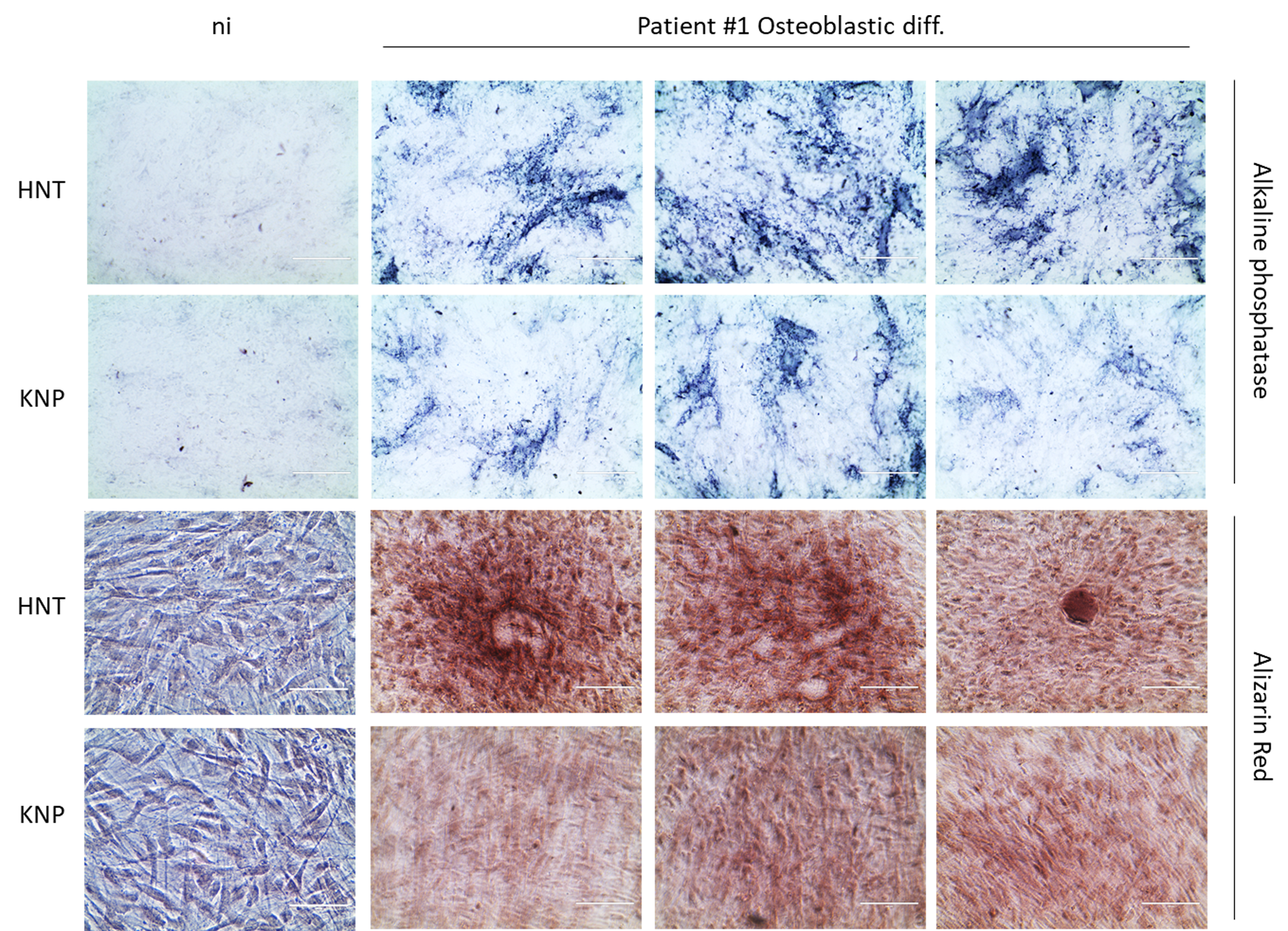

Supplement: Supplementary file 1 [file ijms-23-13214-s001.zip › S2A.tif]

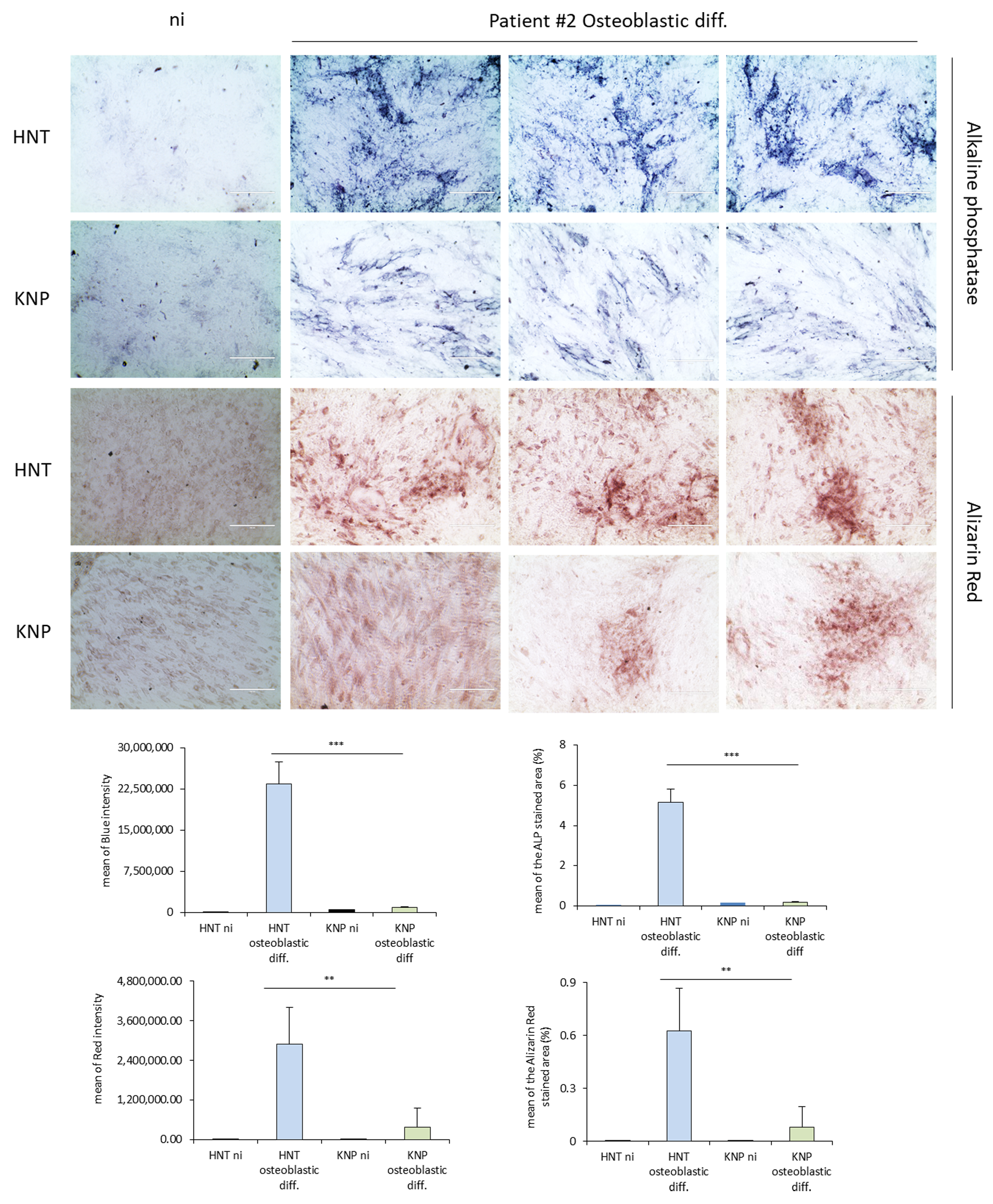

Supplement: Supplementary file 1 [file ijms-23-13214-s001.zip › S2B.tif]

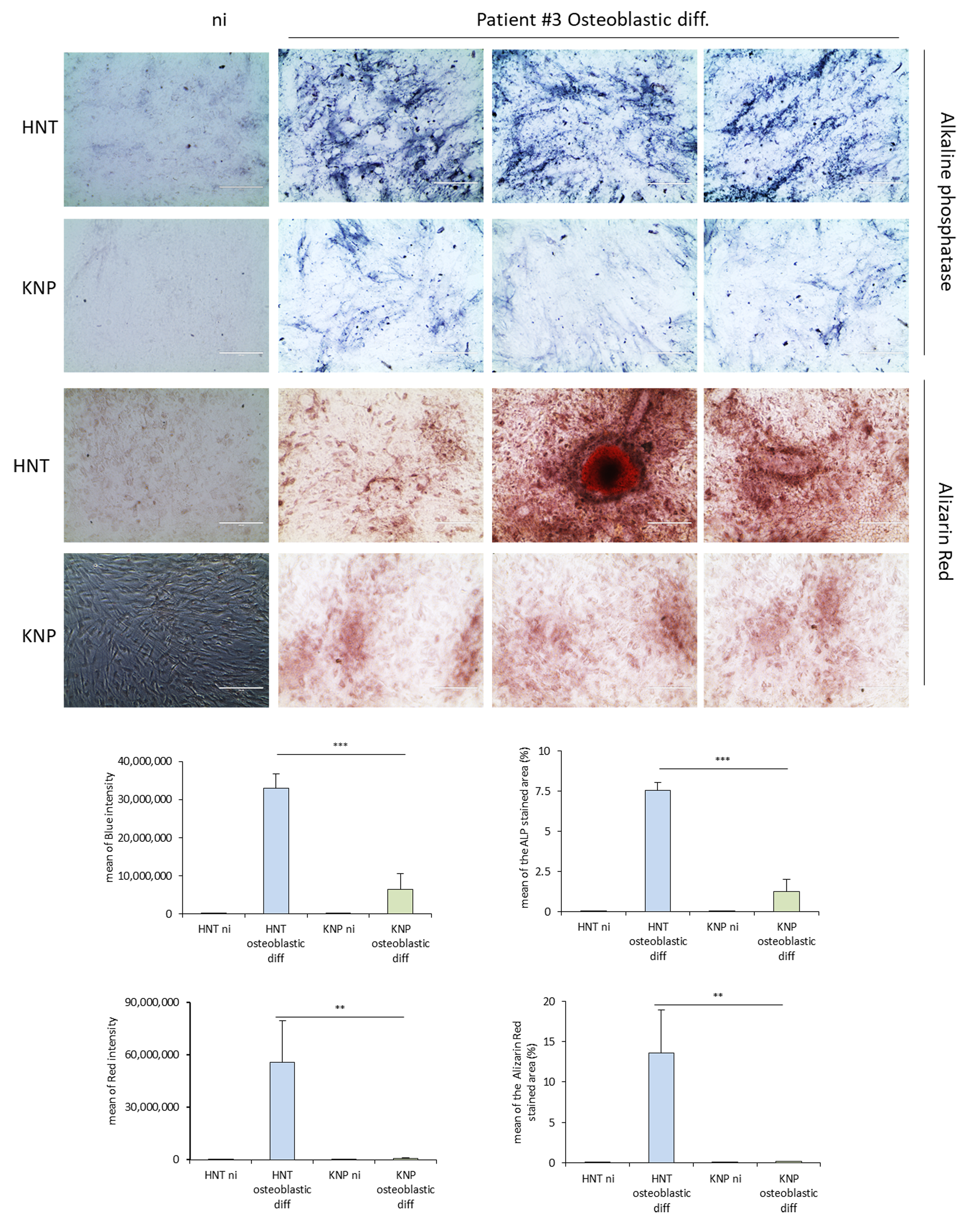

Supplement: Supplementary file 1 [file ijms-23-13214-s001.zip › S2C.tif]
